# Management of a Patient with Cardiovascular Disease Should Include Assessment of Primary and Secondary Immunodeficiencies: Part 1—Primary Immunodeficiencies

**DOI:** 10.3390/healthcare12191976

**Published:** 2024-10-04

**Authors:** Katarzyna Napiórkowska-Baran, Agata Doligalska, Magdalena Drozd, Marta Czarnowska, Dariusz Łaszczych, Marcin Dolina, Bartłomiej Szymczak, Oskar Schmidt, Zbigniew Bartuzi

**Affiliations:** 1 Department of Allergology, Clinical Immunology and Internal Diseases, Collegium Medicum Bydgoszcz, Nicolaus Copernicus University Torun, 85-067 Bydgoszcz, Poland; zbartuzi@cm.umk.pl; 2 Student Research Club of Clinical Immunology, Department of Allergology, Clinical Immunology and Internal Diseases, Collegium Medicum Bydgoszcz, Nicolaus Copernicus University Torun, 85-067 Bydgoszcz, Poland; doli285@wp.pl (A.D.); magdalena.drozd515@wp.pl (M.D.); czarnowska.marta@onet.pl (M.C.); laszczychdariusz@gmail.com (D.Ł.); melminio@wp.pl (M.D.); bartlomiej.szymczak1@gmail.com (B.S.); okischmidt@wp.pl (O.S.)

**Keywords:** inborn errors of immunity, primary immunodeficiencies, secondary immunodeficiencies, immune defects, cardiovascular diseases, heart diseases

## Abstract

Background: Cardiovascular diseases are some of the most prevalent chronic diseases that generate not only high social but also economic costs. It is becoming increasingly crucial to take into account inborn errors of immunity (IEIs, formerly known as primary immunodeficiencies (PIDs)) and secondary immunodeficiencies (SIDs) in the diagnostic and therapeutic management of cardiac patients. The number of diseases classified as IEIs is on the rise, with a current total of 485. It is essential to pay attention not only to already confirmed conditions but also to symptoms suggestive of immunodeficiencies. Objectives: The aim of this article is to present IEIs with cardiovascular symptoms that may cause or exacerbate cardiovascular disease, as well as diagnostic and therapeutic procedures. Results: It is becoming increasingly evident that immunodeficiencies can be responsible for certain cardiovascular conditions, their hastened progression, and difficulties in their control. Conclusions: Early detection of deficiencies improves not only the quality and longevity of patients, but also allows for better control of cardiovascular diseases and even prevention of their occurrence.

## 1. Introduction

Cardiovascular diseases represent a significant public health concern, contributing not only to social costs but also to significant economic burdens. It is, therefore, imperative to consider inborn errors of immunity (IEIs, formerly primary immunodeficiencies (PIDs)) and secondary immunodeficiencies (SIDs) when diagnosing and introducing treatment in cardiac patients. These are groups of diseases with a wide range of underlying symptoms. With consecutive updates from the International Union of Immunological Societies (IUIS), the number of disease entities classified as IEIs has been steadily increasing and is now reaching 485. It is crucial to be vigilant not only for already confirmed conditions but also for symptoms suggestive of immunodeficiencies [1]. Early detection of deficiencies not only improves the quality and longevity of patients but also allows for better control of cardiovascular diseases and even their prevention.

Primary immunodeficiencies unfortunately remain an overlooked issue by physicians across various specialties. This is primarily due to insufficient knowledge on the subject. There is often a misconception that IEIs only affect children, which is not true. Some IEIs present their first symptoms only in adulthood. Such deficiencies include common variable immunodeficiency (CVID), selective IgA deficiency, and IgG subclass deficiency [2]. Research conducted by Boyle JM revealed that 25–40% of IEI cases were diagnosed in patients over 18 years old [3]. Current statistics are even less optimistic, with estimates suggesting that more than 50% of IEI cases worldwide involve adults [2]. Unfortunately, a significant proportion of these diagnoses result from the failure to establish the correct diagnosis in childhood. Other reasons include IEI phenocopies, lack of awareness about the diverse symptoms of IEIs, and the belief that deficiencies are often associated with other genetic defects, manifesting as anomalies in physical examinations, mainly dysmorphisms. Additionally, there is a concerning trend in modern medicine. Rapid advancements have led to patients with diseases of specific organs and systems being treated by particular specialists, resulting in a lack of a holistic view of the patient.

Unfortunately, the current estimated delay from the onset of first symptoms to the diagnosis of IEIs is 16.1 years [4]. For the most common group of IEIs, primary antibody deficiencies, the estimated delay from the onset of first symptoms to diagnosis is 6–12 years [5]. It is rare for a cardiologist to be the specialist who refers a patient for IEI diagnostics.

Diller GP et al. presented results regarding the incidence of increased susceptibility to infections (ISI) or confirmed immune deficiency syndromes (IDS) in patients with congenital heart disease (CHD) and compared them with a control group of the same age without congenital defects [6]. The study included a total of 54,449 patients with CHD. Among them, 14,998 (27.5%) had ISI, and 3034 (5.6%) had documented IDS (compared to 2.9% of the general population of the same age). The analysis showed that IDS was an independent prognostic factor for all-cause mortality. Additionally, ISI and confirmed IDS were associated with a significantly higher risk of emergency hospital admissions during follow-up. Furthermore, the study emphasized that immune system dysfunction is common in patients with CHD and is associated with an increased risk of morbidity and mortality. It also highlighted the need for structured screening for IDS and collaboration with immunology specialists, as immune deficiencies may be amenable to specific treatments. Moreover, further research is needed to assess whether patients with IDS could benefit from intensified antibiotic protection or tailored prophylaxis.

In the study conducted by the author of this paper, an attempt was made for the first time to assess the cardiovascular risk in patients with IEIs presenting with a defect in antibody production [7]. Primary antibody deficiencies are the most prevalent group of IEIs and account for 50–60% of all deficiencies. The results of the study were not promising. A total of 25.5% had cardiovascular disease (mainly hypertension, 18%), 10.5% smoked, 17% were overweight, 14% were obese, and 15% were underweight. Additionally, elevated blood pressure was found in 6.5% of the patients. Lipid metabolism disorders were found in 72.5% of the study cohort. The mean number of risk factors was 5 ± 3 for the entire population and 4 ± 2 for those under 40. A significant proportion of patients (74.5%) had never undergone an echocardiogram. The provided examination revealed that 30% of subjects exhibited abnormalities within the normal range, predominantly in the form of regurgitation (92.5%). In contrast, new pathologies were identified in 28% of the patients. Consequently, it is of paramount importance to prioritize prevention in patients with PADs in order to reduce their cardiovascular risk.

The International Union of Immunological Societies (IUIS), taking into account both genetic background and phenotypic characteristics, has distinguished 10 categories of IEIs (Table 1) [8]. The diagnostic and therapeutic procedure is presented later in the article.

## 2. Cardiovascular Diseases Concomitant with IEI

The coexistence of cardiovascular diseases with IEIs can lead to serious clinical complications. On the one hand, improper functioning of the immune system may contribute to the development of chronic inflammatory conditions, which play key roles in the pathogenesis of many cardiovascular diseases, such as atherosclerosis or coronary artery disease. On the other hand, the presence of cardiovascular diseases may negatively affect the overall health of patients with IEIs, leading to a deterioration in their quality of life and an increased risk of mortality.

In the scientific literature, there are only a handful of reports concerning the relationship between IEIs and cardiovascular diseases. However, comprehensive epidemiological data and a detailed understanding of the pathogenic mechanisms that could explain how these two groups of disorders interact are still lacking. The complexity of the interactions between immune system disorders and heart diseases remains largely unexplored, which constitutes a significant gap in the current medical knowledge.

The authors of this study hope that this article will serve as a catalyst for further research in this area, contributing to a deeper understanding of this issue. Gaining more insight into the links between IEIs and cardiovascular diseases could be crucial for developing new diagnostic and therapeutic strategies, ultimately improving the quality of life and treatment outcomes for patients affected by these conditions.

Inborn errors of immunity with cardiovascular symptoms are presented in Table 2.

## 3. Characteristics of Selected IEIs with Cardiovascular Manifestations

The characteristics of selected IEIs with cardiovascular manifestations are presented below.

### 3.1. Common Variable Immunodeficiency

Common variable immunodeficiency represents the most significant symptomatic congenital immunodeficiency. It has an estimated prevalence of 1:25,000 in adult patients. Individuals with CVID exhibit an elevated susceptibility to infections, particularly those affecting the respiratory tract [9,17]. Additionally, they are prone to autoimmune or inflammatory complications, including immune thrombocytopenia, interstitial lung disease, and enteropathies. CVID presents with a variety of clinical manifestations, all of which are characterized by low levels of IgG, IgA, and a poor response to vaccination. A monogenic cause of CVID is identified in only 10–20% of patients. The prognosis of CVID patients with associated inflammation is not favorable [20].

A study conducted by Mattila J et al. demonstrated that patients with CVID are more likely to be diagnosed with atherosclerotic cardiovascular disease, encompassing ischemic heart disease, peripheral vascular disease, and cerebrovascular disease. The prevalence of cardiovascular disease in the group with CVID was 21.69%, while in the control group comprising randomly selected patients without CVID, it was 9.55% [26]. A multivariate analysis revealed that CVID is an independent risk factor for atherosclerotic cardiovascular disease. Patients may present with endocarditis, myocarditis, or pericarditis, which are associated with increased susceptibility to infection and pulmonary hypertension as a complication of pulmonary conditions [9,23,27].

### 3.2. Hyper IgE Syndrome

Hyper IgE syndrome is characterized by eczema, increased susceptibility to infections of the skin and upper and lower respiratory tracts with increased levels of IgE in the body. There are two types: autosomal dominant hyper-IgE syndrome (AD-HIES) and autosomal recessive hyper-IgE syndrome (AR-HIES). In AD-HIES, abnormalities in the immune, vascular, skeletal systems, and abnormalities in connective tissue development are observed. In AR-HIES, there are no changes in the musculoskeletal system, but there are infections of the skin, lungs, and central nervous system [10]. HIES is mainly caused by a mutation in the STAT3 gene. The STAT3 molecule is involved in immune-mediated processes, healing, and non-immune mechanisms, which explains symptoms from other systems [11]. A mutation in this gene interferes with the modulation of IgE production in B lymphocytes by IL-6, IL-10, and IFN-gamma. This leads to excessive production of IgE and impaired neutrophil chemotaxis. IL-6 plays a crucial role in the synthesis of TH17 cells, so its deficiency results in a reduction in the levels of these lymphocytes. Consequently, the immune system is unable to effectively combat pathogens. Vascular manifestations include anomalies that can result in coronary, cerebral, and aortic aneurysms. Vascular aneurysms may be complicated by myocardial infarction, while cerebral aneurysms can be complicated by lacunar infarctions. IgE levels in patients with HIES are often elevated from birth. This can be explained by their correlation with the occurrence of congenital persistent venous duct in these patients [12]. The aforementioned pathologies suggest an integral role of STAT3 in vascular remodeling.

Additionally, the presence of obstruction in small arterial vessels, reduced perfusion in large vessels, and leukocytoclastic vasculitis were also observed. In these patients, hypereosinophilia was observed, which, when combined with ongoing inflammation and defective angiogenesis, may contribute to the development of vascular-related disorders [18].

### 3.3. Complement Component Deficiency

C1 inhibitor deficiency is also referred to as hereditary angioedema. The dilation of blood vessels is a consequence of increased diastasis in smooth muscle, which is caused by uncontrolled bradykinin production. Edema can affect the hands, feet, gastrointestinal tract, and respiratory system. Involvement of the upper and lower respiratory tracts, especially the larynx, can be complicated by respiratory distress [19]. C1 esterase inhibitor suppresses bradykinin production by inhibiting kallikrein and factor XIIa. In the absence of this inhibitor, bradykinin levels increase, leading to edematous reactions [14]. 

C2 deficiency may present as either an asymptomatic condition or as a severe infection with encapsulated bacteria. C2 deficiency may be accompanied by autoimmune diseases, such as systemic lupus erythematosus. Two main types of deficiency have been identified: type I, which is characterized by a lack of C2 synthesis, and type II, which is associated with a blockade of C2 secretion. It participates in the mechanism of antibody-dependent complement activation and in the lectin pathway of innate immunity. Researchers from Sweden have observed a correlation between C2 deficiency and cardiovascular disease. In their patient population, they observed an increased frequency of cardiovascular incidents, including myocardial infarction, aortic dissection, and congestive heart failure. As reported by Jönsson et al., the risk of myocardial infarction is four times higher than that observed in the general Swedish population [15]. The complement system contributes to the activation and modulation of chronic inflammation. A deficiency of the complement component C2, involved in the lectin pathway, may be responsible for the inflammatory component in the development of atherosclerosis. This provides an explanation for the increased cardiovascular risk in patients with this IEI, given the documented importance of atherosclerosis in this process [16].

### 3.4. Chronic Mucocutaneous Candidiasis

Signal transducer and activator of transcription 1 (STAT1) gain-of-function mutations are considered to be the cause of most hereditary chronic mucocutaneous candidiasis (CMC). The frequency of its identification has increased along with the phenotypic diversity under which it is concealed. The broad spectrum of conditions includes infections, autoimmune diseases, autoinflammatory diseases, vascular aneurysms, and malignancies [28,29]. The underlying mutation is the coiled-coin or DNA-binding domain of the gene in immune cells. Initially, gain-of-function was attributed to an increase in STAT1 phosphorylation following stimulation with type I and II interferons (IFN) and IL-27. Subsequently, it was demonstrated that delayed STAT1 dephosphorylation in response to IFN and IL-27 occurs, and that STAT1 protein levels can increase with normal dephosphorylation [21]. This results in the inhibition of IL-12R/IL-23R signaling, which in turn leads to an impaired Th1/Th17 response, thus increasing susceptibility to fungal infections [22].

It has been demonstrated that CMC is associated with an increased incidence of brain aneurysms [13]. A case report described a patient who died as a result of a ruptured basilar artery aneurysm. A postmortem examination revealed Candida fragments in this artery. The inadequate T-lymphocyte response to fungal infection, in this case, resulted in the rapid and intense expansion of pathogens in the body. A similar case was observed in a patient with CMC and diagnosed idiopathic myocarditis [30]. The prolonged inflammatory fungal process caused complicated myocardial fibrosis, ultimately leading to the patient’s fatal arrhythmia. At the time of diagnosis, an ischemic and drug-induced etiology was deemed unlikely. Treatment was focused on the autoimmune approach of the inflammation, and resolution of the actively ongoing inflammation was achieved.

### 3.5. DiGeorge Syndrome

DiGeorge syndrome (DGS) is a disease with a broad phenotypic spectrum. The etiology of DGS is attributed to the abnormal development of the pharyngeal pockets, which is a consequence of a microdeletion of chromosome 22. Symptoms include cardiac anomalies (CAs), recurrent infections, facial dysmorphia, hypoplasia or aplasia of the thymus, cleft palate, developmental delay, and accompanying immunodeficiency [31]. Co-occurring CAs include tetralogy of Fallot, a common trunk artery, an interrupted aortic arch, a ventricular septal defect, and an atrial septal defect (ASD). The precise prevalence of congenital heart defects in DGS is challenging to ascertain, given the inconsistencies in the literature. Nevertheless, the available evidence suggests a substantial prevalence, with estimates ranging from 48.5% to 80% of DGS patients with CAs, contingent on the specific study population. In addition to DGS, numerous cardiopulmonary complications have been observed in patients with tetralogy of Fallot and pulmonary atresia. These include heart failure, severe respiratory failure, stridor, pneumonia, and sepsis. A substantial body of evidence suggests that these patients have a higher mortality rate. Furthermore, the pulmonary arteries of these patients were found to be hypoplastic in comparison to patients without the deletion [32]. The presence of undiagnosed and untreated IEIs has been demonstrated to exacerbate the course of concomitant cardiac disorders. This illustrates the importance of prenatal screening, particularly in the context of heart defects. It is recommended that children and adults with dysmorphic features characteristic of DGS are screened. An accurate diagnosis allows the child’s parents to be prepared for potential surgical intervention. Unplanned cardiac surgery can be planned with the possibility of predicting the need for surgery. Subsequent postoperative complications, including coronary syndromes, may be associated with more frequent reoperations, use of a ventilator, a worse course, and a longer recovery period.

### 3.6. Severe Congenital Neutropenia Type 4

Severe congenital neutropenia type 4 (SCN type 4) is induced by a mutation in the G6PC3 gene, which encodes 3 glucose-6-phosphatase-beta. Consequently, neutropenia and neutrophil dysfunction occur as a result of impaired actin assembly, CD11b expression, and superoxide anion production [33]. A reduction in cell phagocytosis capacity is associated with a decline in bactericidal activity and an extended course of infection. The majority of patients present with congenital heart defects, increased superficial venous visibility (referred to as IVSV), inflammatory bowel disease, and congenital urogenital malformations. Congenital defects are most commonly ASDs, heart murmurs, and peripheral venous insufficiency. As observed by López-Rodríguez L et al., ASDs typically manifest as quiet murmurs, which can result in its prolonged unnoticed presence [24]. Nevertheless, when diagnosed at a later age, they are found to be associated with surgical intervention. The formation of iatrogenic ventricular septal defects is likely to occur with age. The cause of this condition is believed to lie in impaired embryogenesis, resulting from a defect in the G6PC3 gene. Additionally, cases of pulmonary hypertension and cardiac arrhythmias, including congenital Wolff–Parkinson–White syndrome, have been documented. Furthermore, this condition has been diagnosed in young adults and identified as a potential cause of sudden death [25]. The prevention and early diagnosis of cardiac disorders can prevent the occurrence of sudden and chronic complications.

### 3.7. Autoinflammatory Diseases

Autoinflammatory diseases (ADs) are a group of diseases with developing inflammation in the absence of pathogens or autoantibodies. Inflammasomes, which are components of the innate immune system, play a vital role in the pathogenesis of these diseases. Initially distinguished only on the phenotypically common fever, familial Mediterranean fever, tumor necrosis factor receptor-associated periodic syndrome (TRAPS), hyper-IgD syndrome (HIDS), cryopyrin-associated periodic syndrome (CAPS), and PFAPA syndrome were distinguished [34]. The latter is manifested as a periodic fever with aphthous stomatitis, pharyngitis, and lymphadenitis. Gradually, the group of autoinflammatory diseases was expanded based on pathophysiological and molecular changes [35]. In addition to fever, symptoms commonly associated with this condition include skin rashes, joint pain, and the inflammation of serous membranes [36]. On the cardiac side, pericarditis is a significant consideration. Increased incidence of heart failure, valvular heart disease, and myocardial infarction could be observed in CAPS syndrome. One of the most frequently affected organs in CAPS is the heart. This was demonstrated by Pons I et al. in a study comprising more than 700 patients, of whom over half had serious cardiac disease [37]. Significant observations were made in the incidence of heart failure, valvular heart disease, and myocardial infarction. Nevertheless, this was not associated with an increased mortality rate. Furthermore, pericarditis represents a significant consideration. This information may have a significant impact on the treatment of cardiovascular disease that develops later in life in patients with AD. It is possible that early recognition may subsequently contribute to the implementation of prompt lifestyle modifications, dietary changes, and the initiation of a suitable treatment in an effort to prevent a prevalent complication, such as myocardial infarction.

## 4. Diagnostics of IEIs

When diagnosing IEIs, the patient’s history and consideration of potential deficiencies are crucial. Clinical immunology practice over the years has shown that IEI diagnoses are often made by physicians who have previously encountered IEI patients or have actively sought to enhance their understanding of these conditions. Fortunately, awareness of IEIs among physicians has been gradually increasing, as evidenced by the fact that the majority of IEI diagnoses have occurred within the last decade (up to 70% in the authors’ study) [38].

It is inevitable that every physician, regardless of specialization, will encounter a patient with an IEI during their career. In some cases, it is the cardiologist who first identifies an IEI, especially when the patient presents with endocarditis, myocarditis, or pericarditis. The fundamental aspect of diagnosing an IEI is considering its possibility.

On the Jeffrey Modell Foundation website, we found a study to help in recognizing IEIs, i.e., it provides 10 warning signs suggesting primary immunodeficiency in both children and adults [39]. The relatively high accuracy of the signs devised by the researchers enabled IEIs to be diagnosed with a sensitivity of 56% [40]. These are presented in Table 3. A study by Dąbrowska A et al. demonstrated that when four additional symptoms, including severe eczema, allergies, hematologic and oncologic disorders, and autoimmunity, are incorporated into the aforementioned list, the frequency of IEI diagnoses significantly increases [41].

In order to diagnose IEI, the diagnostic scheme presented in Table 4 is applicable, outlining the diagnosis of primary antibody deficiency, which accounts for 50–60% of IEIs and rarer IEIs [42]. The table developed by the authors provides a comprehensive diagnostic guide for IEI intended for physicians who are not clinical immunologists.

The key to diagnosing IEIs is to recognize them as early as possible and refer them to a clinical immunologist to verify the diagnosis, establish recommendations, and provide appropriate treatment. Remember that it is cooperation between specialists that provides the greatest chance for optimal treatment of patients.

In patients with antibody production disorders, diagnostic methods based on their determination may be non-diagnostic. This is of particular importance in determining the etiological factor, e.g., myocarditis. In people taking immunoglobulins, it should be remembered that they are prepared from a pool of plasma from at least 1000 donors. In recipients of such preparations, IgG antibodies (e.g., anti-HCV, ANA) may be the result of the presence of antibodies from immunoglobulin donors. A certain diagnosis can be made only 4–6 weeks after discontinuing the preparation, provided that the production of antibodies in this class is not disturbed.

## 5. Therapeutic Management in IEIs

Helpful in the care of patients with IEIs are the published protocols for managing patients with primary antibody deficiencies, which, as previously mentioned, constitute the most common group of immune deficiencies. These protocols include medical history, recommended tests, and the frequency of their performance. A summarized version of the protocols is presented in Table 5. Due to the fact that secondary deficiencies often occur in patients with IEIs, they have also been included. In patients with radiosensitivity, radiological examinations (X-ray, CT) are contraindicated, except in cases of direct threat to life. Ultrasound and NMR are recommended. Radiotherapy is also contraindicated. Diseases associated with radiosensitivity include severe combined immunodeficiency (SCID), SCID with hypersensitivity to ionizing radiation, primary immunodeficiencies with impaired DNA repair, and hypersensitivity to ionizing radiation: ataxia-telangiectasia syndrome, Nijmegen syndrome, and RIDDLE syndrome.

Therapeutic management of IEIs should be multidirectional and include personal hygiene, isolation during periods of infection, avoiding exposure to crowds, early treatment of infections, monitoring nutritional status, regular physical activity adapted to age, condition, and health status, and reducing stress. In selected cases, replacement with immunoglobulin preparations (intravenously or subcutaneously) or antibacterial/antifungal/antiviral prophylaxis is necessary. The underestimated role of additional vaccinations should not be forgotten. In case of the need for the transfusion of blood substitutes, the patient should receive irradiated, leukocyte-depleted preparations. In special cases, hematopoietic stem cell transplantation, gene therapy, or enzyme replacement therapy (ERT) is used. We should also not forget about genetic counseling [42].

It is highly relevant to implement additional vaccinations due to their broad effects, benefiting not only immunodeficient individuals but also those in close contact with them (cocoon vaccination). Live vaccines are generally not recommended for patients with cellular immunity disorders and severe humoral immune deficiencies (e.g., XLA, CVID), though each case should be evaluated individually [43]. Mild antibody deficiencies, such as selective IgA deficiency, IgG subclass/y deficiency, or specific antibody deficiency, do not contraindicate the use of live vaccines [44]. Killed (inactivated) vaccines, such as the influenza vaccine, are recommended, along with protein and polysaccharide vaccines (against S. pneumoniae, H. influenzae, meningococcus, and tetanus). The COVID-19 vaccine is also recommended. Vaccination is also beneficial due to the fact that vaccine components containing proteins or polysaccharides of given microorganisms are able to induce a defensive reaction of the organism not only against microorganisms or their components contained in the vaccine, but also against other microorganisms of similar structure, sometimes not closely related to each other [45]. Additionally, vaccines, in addition to inducing specific immunity, also strengthen non-specific immunity to other pathogens, including viruses, fungi, and parasites.

In certain cases, patients may need immunoglobulin preparations. Indications include IgG concentration deficiencies, specific antibody deficiency, and sometimes acute autoimmune diseases. The decision to start treatment is primarily based on the patient’s clinical condition rather than the level of IgG reduction alone. It should be noted that immunoglobulins are derived from a plasma pool of at least 1000 donors. Antibodies of the IgG class (e.g., anti-HCV, ANA) in recipients of these preparations are determined by donor antibodies, and a reliable diagnosis can only be made 4–6 weeks after discontinuation of the preparation, provided antibody production in this class is intact [46].

Chronic drug prophylaxis is necessary for some patients. Infectious complications related to IEIs that require continuous antimicrobial prophylaxis include chronic granulomatous disease, hyper-IgE syndrome, and some IEIs with neutropenia and/or severe chronic/recurrent infections and/or specific complications, like bronchial dilation [47]. Periodic prophylactic antibiotic therapy may be needed during fall and winter, post-tooth extraction, during ENT procedures, or other tissue-disrupting procedures. Prolonged antibiotic therapy might be necessary for infections, such as sinusitis, otitis media, and pneumonia. Microbe-specific prophylaxis is recommended for staphylococci (typically trimethoprim-sulfamethoxazole), streptococci, Mycoplasma, and nontuberculous mycobacteria (usually azithromycin) [48]. Continuous antifungal prophylaxis is required for patients with chronic granulomatous disease, hyper-IgE syndrome, or chronic cutaneous-mucosal candidiasis. Aspergillus infections are generally treated with itraconazole, while Candida infections are treated with fluconazole. Prolonged treatment might be needed for fungal pneumonia or central nervous system mycosis [49]. Continuous antiviral prophylaxis is required for deficiencies, such as SCID and DOCK8. Prolonged therapy, often several months, is needed to prevent infections from herpes simplex virus, VZV, or CMV. Acyclovir is used for HSV and VZV infections, and ganciclovir for CMV infections [48].

Hematopoietic stem cell transplantation is routinely used for severe combined immunodeficiencies, Wiskott–Aldrich syndrome, chronic granulomatous disease, or based on an individual risk-benefit analysis (some other severe PIDs) [50]. Gene therapy, like enzyme replacement therapy, is currently a treatment option available for severe combined immunodeficiency with adenosine deaminase deficiency (ADA-SCID). Encouraging data exist for X-SCID and preclinical work for Artemis-SCID and RAG1-SCID, which pave the way for this therapy to become a viable treatment option [51].

In general, treatment of this group of patients is consistent with the treatment of immunocompetent individuals, using both local and systemic pharmacological agents. However, timely and appropriate treatment is crucial for patients with IEIs to prevent complications and the development of new concomitant diseases.

## 6. Conclusions

Cardiovascular diseases have a profound impact on the entire body. Conversely, diseases of various organs and systems can affect the onset or course of cardiovascular diseases. Consequently, it is imperative to consider immunodeficiencies in the care and treatment of cardiac patients. Unfortunately, we can only find isolated publications concerning the cardiovascular system and inborn errors of immunity.

Congenital immune defects can coexist with secondary deficiencies. When assessing a cardiology patient, it is crucial to evaluate the patient for primary immunodeficiency. Only a holistic evaluation of the patient that considers both primary and secondary immunodeficiencies provides the best opportunity for the optimal treatment of cardiovascular diseases. It is clear that this group of patients requires additional education as well as more frequent examinations and follow-up visits.

## Figures and Tables

**Table 1:** Classification of various IEIs and their subtypes (adapted from [8]). CID—combined immunodeficiency; CVID—common variable immunodeficiency; EBV—Epstein–Barr virus; FHL—familial hemophagocytic lymphohistiocytosis; HPV—human papillomavirus; HSE—herpes simplex encephalitis; SCID—severe combined immunodeficiency; TLR—Toll-like receptor.

Inborn Error of Immunity	Subtypes
Immunodeficiencies affecting cellular and humoral immunity	T-B+ SCID- T-B− SCID CID, generally less profound than SCID
Combined immunodeficiencies with associated or syndromic features	Immunodeficiency with congenital thrombocytopenia DNA repair defects other than those listed in Table 1 Thymic defects with additional congenital anomalies Immuno-osseous dysplasias Hyper IgE syndromes (HIES) Defects of vitamin B12 and folate metabolism Anhidrotic ectodermodysplasia with immunodeficiency (EDA-ID) Calcium channel defects Other defects
Predominantly antibody deficiencies	Severe reduction in all serum immunoglobulin isotypes with profoundly decreased or absent B cells, agammaglobulinemia Severe reduction in at least 2 serum immunoglobulin isotypes with normal or low number of B cells, CVID phenotype Severe reduction in serum IgG and IgA with normal/elevated IgM and normal numbers of B cells, hyper IgM Isotype, light chain, or functional deficiencies with generally normal numbers of B cells
Diseases of immune dysregulation	Familial hemophagocytic lymphohistiocytosis (FHL syndromes) FHL syndromes with hypopigmentation Regulatory T cell defects Autoimmunity with or without lymphoproliferation Immune dysregulation with colitis Autoimmune lymphoproliferative syndrome (ALPS, Canale–Smith syndrome) Susceptibility to EBV and lymphoproliferative conditions
Congenital defects of phagocyte number or function	Congenital neutropenias Defects of motility Defects of respiratory burst Other non-lymphoid defects
Defects in intrinsic and innate immunity	Mendelian susceptibility to mycobacterial disease (MSMD) Epidermodysplasia verruciformis (HPV) Predisposition to severe viral infection Herpes simplex encephalitis (HSE) Predisposition to invasive fungal diseases Predisposition to mucocutaneous candidiasis TLR signaling pathway deficiency with bacterial susceptibility Other inborn errors of immunity related to non-hematopoietic tissues Other inborn errors of immunity related to leukocytes
Autoinflammatory disorders	Type 1 interferonopathies Defects affecting the inflammasome Non-inflammasome-related conditions
Complement deficiencies	Deficiencies of individual components of the complement system
Bone marrow failure	Fanconi anemia Dyskeratosis congenita MECOM deficiency
Phenocopies of inborn errors of immunity	VEXAS (vacuoles, E1 enzyme, X-linked, autoinflammatory, somatic) syndrome Chronic mucocutaneous candidiasis Severe COVID-19

**Table 2:** Cardiovascular diseases in inborn errors of immunity (IEIs) based on works by Tangye et al. and Mohammadi et al. [1,9]. Abbreviations: APDS—activated phosphoinositide 3-kinase δ syndrome; CAPS—cryopyrin-associated periodic syndromes; CHARGE—coloboma, heart defect, atresia of the choanae, retardation, genital, and ear abnormalities syndrome; CMC—chronic mucocutaneous candidiasis; CVID—common variable immunodeficiency; DGS—DiGeorge syndrome; DOCK8—dedicator of cytokinesis protein 8; G6PC3—glucose-6-phosphatase enzyme deficiency; GOF—gain-of-function; HIES—hyper-IgE syndrome; IFNAR1—interferon alpha and beta receptor subunit 1; LRRC8—leucine-rich repeat-containing protein 8; MST1/STK4—serine/threonine-protein kinases 4; MWS—Muckle–Wells syndrome; NISBD—neonatal inflammatory skin and bowel disease; NOMID—neonatal onset multisystem inflammatory disease; PNP—purine nucleoside phosphorylase; SCN—severe congenital neutropenia; STAT1/STAT3—signal transducer and activator of transcription 1/3; TLR3—Toll-like receptor 3; TRAPS—tumor necrosis factor receptor-associated periodic syndrome; WHIM—warts, hypogammaglobulinemia, immunodeficiency, myelokathexis; XLA—X-linked agammaglobulinemia.

Cardiovascular Manifestation	Associated IEIs
Aneurysms	STAT1 GOF, all subtypes of HIES, CMC [8,9,10,11,12,13]
Angioedema	C1 inhibitor, factor XII deficiencies [9,14]
Atherosclerosis (increased risk)	C2 deficiency, CVID [15,16]
Congenital heart disorder	DGS, SCN type 4, CHARGE syndrome, Kabuki Syndrome, G6PC3, MST1/STK4 deficiencies, Shokeir syndrome, Stoll syndrome, orotic aciduria, Timothy syndrome, TIIAC syndrome, asplenia syndrome, ring chromosome 21, deletion of long arm of chromosome 2 (2q37), LRRC8, WHIM, MECOM deficiency [2,8,9]
Congenital patent ductus venosus	All subtypes of HIES [17,18,19]
Coronary artery tortuosity or dilation	STAT3-mutated HIES [9,11,12]
Endocarditis	STAT3-mutated HIES, CVID [9,20]
High cutoff level of peak systolic aortic pressure	CVID [20]
Hypertension	Selective IgE deficiency [9]
Interrupted aortic arch	DGS [21,22]
MISC after COVID-19	IFNAR1 deficiency [8]
Murmur or cyanosis	DGS, SCN type 4, CHARGE syndrome, Kabuki Syndrome, MST1/STK4 deficiency [8,9]
Myocarditis	CVID, STAT1 GOF, PNP deficiency, NISBD, Aicardi–Goutieres syndrome, TLR3 deficiency [8,20,23]
Pericarditis	CVID, APDS, DOCK8 deficiency, XLA, Mulibrey nanism (constrictive), autoinflammatory disorders (CAPS-MWS, CAPS-NOMID, TRAPS), Waldmann’s disease, Blau syndrome [8,9]
Pseudoaneurysms	All subtypes of HIES [8,9,11,12]
Pulmonary arterial hypertension	CVID [9,17]
Superior vena cava syndrome	All subtypes of HIES [8,9,11,12]
Thrombosis	All subtypes of HIES [8,9,11,12]
Valvular regurgitation	CVID [9,17]
Venous angiectasis	G6PC3 deficiency [24,25]

**Table 3:** The Jeffrey Modell Foundation’s 10 warning signs of primary immune deficiency [35].

**Warning Signs in Adults**
≥2 new ear infections within 1 year. ≥2 new sinus infections within 1 year, in the absence of allergy. 1 case of pneumonia per year for >1 year. Chronic diarrhea with weight loss. Recurrent viral infections (colds, herpes, warts, condyloma). Recurrent need for IV antibiotics to clear infections. Recurrent, deep abscesses of the skin or internal organs. Persistent thrush or fungal infection on skin or elsewhere. Infection with normally harmless tuberculosis-like bacteria. A family history of PID.
**Warning Signs in Children**
≥4 new ear infections within 1 year. ≥2 serious sinus infections within 1 year. ≥2 months on antibiotics with little effect. ≥2 cases of pneumonia within 1 year. Failure of an infant to gain weight or grow normally. Recurrent, deep skin or organ abscesses. Persistent thrush in mouth or fungal infection on skin. Need for IV antibiotics to clear infections. ≥2 deep-seated infections including septicemia. A family history of PID.

**Table 4:** Diagnostic scheme for inborn errors of immunity.

Medical Interview	
➢ Recurrent/prolonged/chronic/opportunistic/unusual infections. ➢ Autoimmunity. ➢ Hematological disorders. ➢ Neoplasms. ➢ Allergies (unusual and/or difficult to treat).	
rule out secondary immunodeficiencies	
antibody production disorders suspected	
➢ if possible, perform the following:	
Morphology with manual smear	Assesses the number of WBCs and lymphocytes.
Proteinogram with evaluation of total protein and γ-globulin fraction	Can detect protein loss and secondary antibody deficiency; Immunoglobulins are mainly contained in the γ-globulin fraction (all immunoglobulin classes) and to a small extent in the β-globulin fraction (IgA and IgM).
Determine concentrations of major immunoglobulin classes IgA, IgG, IgM	The tests are available at any commercial laboratory and most hospitals; The cost of the test is relatively modest in comparison to other specialized tests and examinations that patients may perform independently; It is crucial to note the distinct norms that pertain to specific age groups; Remember, antibodies (i.e., immunoglobulins) are produced only by B lymphocytes.
Lymphocyte phenotyping	Allows for the determination of the number of B lymphocytes.
antibody production disorders suspected ➢ refer the patient to an immunology clinic	
cellular immune disorders suspected	
➢ if possible, perform the following:	
Morphology with manual smear	Assesses lymphocyte count.
Lymphocyte phenotyping	Allows for the determination of the number of T lymphocytes (T, Th, Tc),
cellular immune disorders suspected refer the patient to an immunology clinic	
phagocytosis disorders suspected	
if possible, perform the following:	
Morphology with manual smear	Assesses neutrophil count.
Phagotest and Bursttest	Assesses respiratory burst.
phagocytosis disorders suspected refer the patient to an immunology clinic	
disorders of the complement system suspected	
if possible, perform the following:	
CH50, C3,C4	Determines the functional status of the classical complement pathway.
AH50	Determines the status of the alternative complement pathway.
disorders of the complement system suspected refer the patient to an immunology clinic	

**Table 5:** Protocol for the care of a patient with cardiovascular disease and coexisting primary immunodeficiency, taking into account secondary immunodeficiency (adapted from [42]).

Protocol for the care of a patient with cardiovascular disease and coexisting immunodeficiency	
Last name and first name	
Year of fulfillment	
Date of birth/age	
Diagnosis of immunodeficiency (descriptively + ICD-10)	
Information about the immunologist taking care of the patient Phone number Mail	
Height (cm)	
Bodyweight (kg)	
BMI and nutritional status score: Malnutrition (<18.5) Normal nutrition (18.5–24.9) Overweight (25.0–29.9) Obesity (≥30)	
Stimulants: Alcohol Drugs Medicines Other	
Vaccinations (in the case of indications or contraindications to vaccinations, this should also be included in this point, as well as information on the vaccinations received thus far)	
Contraindication to radiological examinations (X-ray, CT scan)	YES/NO
Dentition:	Normal/abnormal Teeth to be treated
Echocardiogram result	Normal/abnormal Recommended frequency of check-ups:
Medical history for secondary immunodeficiencies Chronic diseases: Type 2 diabetes mellitus Chronic kidney disease Rheumatological disease Inflammatory bowel disease Other autoimmune disease Neoplasms Chronic infections Other Medicaments: Immunosuppressive medications Cardiotoxic medications Other Suspected deficiency of non-nutritive bioactive compounds	YES/NO YES/NO YES/NO YES/NO YES/NO YES/NO YES/NO YES/NO YES/NO YES/NO YES/NO YES/NO YES/NO YES/NO
Other cardiovascular risk factors Lipid metabolism disorders Sedentary lifestyle Other	YES/NO YES/NO YES/NO YES/NO

## Data Availability

No new data were created or analyzed in this study. Data sharing is not applicable to this article.
